# Global thermal tolerance compilation for freshwater invertebrates and fish

**DOI:** 10.1038/s41597-025-05832-w

**Published:** 2025-08-26

**Authors:** Helena S. Bayat, Fengzhi He, Graciela Medina Madariaga, Camilo Escobar-Sierra, Sebastian Prati, Kristin Peters, Jonathan F. Jupke, Jurg W. Spaak, Alessandro Manfrin, Noel P. D. Juvigny-Khenafou, Xing Chen, Ralf B. Schäfer

**Affiliations:** 1 Faculty of Biology, University of Duisburg-Essen, Essen, Germany; 2 Research Center One Health Ruhr, University Alliance Ruhr, Essen, Germany; 3 State Key Laboratory of Black Soils Conservation and Utilization, Key Laboratory of Wetland Ecology and Environment, Northeast Institute of Geography and Agroecology, Chinese Academy of Sciences, Changchun, China; 4 Leibniz Institute of Freshwater Ecology and Inland Fisheries, Berlin, Germany; 5 Geography Department, Humboldt-Universität zu Berlin, Berlin, Germany; 6 University of Cologne, Institute of Zoology, Cologne, Germany; 7 Aquatic Ecology, Faculty of Biology, University of Duisburg-Essen, Essen, Germany; 8 Centre for Water and Environmental Research (ZWU), University of Duisburg-Essen, Essen, Germany; 9 Department of Hydrology and Water Resources Management, Institute for Natural Resource Conservation, Kiel University, Kiel, Germany; 10 Institute for Environmental Sciences iES, RPTU Kaiserslautern-Landau, Landau, Germany; 11 Institute of Aquaculture, University of Stirling, Scotland, UK; 12 Institute of Biology, Freie Universität Berlin, Berlin, Germany

**Keywords:** Freshwater ecology, Ecophysiology, Limnology, Freshwater ecology, Climate-change ecology

## Abstract

Scientists have investigated the thermal tolerance of organisms for centuries, yet the field has not lost relevance as the environmental threats of thermal pollution and global change sharpen the need to understand the thermal vulnerability of organisms in landscapes increasingly subjected to multiple stressors. Freshwater fish and invertebrates are greatly underrepresented in recent large-scale compilations of thermal tolerance, despite the importance of freshwaters as a crucial resource and as havens for biodiversity. Therefore we compiled ThermoFresh, a thermal tolerance database for these organisms that includes literature from 1900 until the present, sourced from five languages to counteract geographic bias. The database contains over 6800 records for over 900 species, including 470 invertebrates, as well as 505 thermal tolerance tests conducted with additional stressors present. We provide a valuable resource to test hypotheses on thermal risks to freshwater organisms in present and future environments subject to multiple stressors.

## Background & Summary

Thermal limits of life have interested researchers since at least the 1700s^1–5^, with early comparisons of thermal tolerances between organisms published in the late 1800s^3^. Despite the large volume of research on the biological and ecological influence of temperature in the last two centuries, many questions regarding thermal tolerances remain unanswered^6–8^. For example, the relationship between oxygen and thermal tolerance is still contested^9,10^, and the influence of thermal tolerance on range limits not fully understood^11,12^. The evolutionary determinants of thermal tolerance continue to engage contemporary research^13–17^.

Furthermore, thermal tolerance can be measured in different ways, complicating large-scale comparisons^18–22^. Two fundamental methodologies have emerged to measure thermal tolerance of a species in the last century. One method involves ramping the temperature until a specified endpoint is reached, known as the critical thermal method, coined by Cowles and Bogert in 1944^23^, though similar methods existed previously^24,25^. The metrics for upper and lower thermal tolerance are known as critical thermal maximum (CTmax) and critical thermal minimum (CTmin), respectively, which represent the upper and lower bounds of a thermal performance curve^26–28^. The most commonly measured endpoint for this method is the loss of equilibrium, which represents a temperature at which an organism can no longer escape conditions that would lead to death; however, the organism should be able to recover once removed from the test^19,29^. This method has gained popularity over time, as the test can be conducted quickly, without permanently harming the organisms, repeatedly on the same organism, and with a small sample of test organisms^29^. Modern mobile heating units allow this methodology to be conducted in the field^30,31^. The lethal thermal maximum (LTmax) or lethal thermal minimum (LTmin) metrics are reached when the temperature is ramped until death occurs^32^. The second method is a static assay, wherein groups of organisms are kept at a fixed temperature for a fixed period of time. This method was formally named the incipient lethal method by Fry in 1947^33^, based on work by others in the two decades prior^34–38^. Percentage mortality is assessed as an endpoint for each group, and statistical methods such as probit analysis used to evaluate the temperature at which a certain percentage of the group is affected^37^. The LT50 metric is the temperature at which 50% of the organisms perish, analogous to the LC50 of ecotoxicological assays for chemicals^33,39^. As with LC50 metrics, LT50 metrics are commonly measured at 24, 48, or 96 hours^18^. The incipient upper or lower lethal temperature (IULT and ILLT, respectively) is a metric distinguished from the LT50 by the longer time course of the test – the IULT represents the temperature which is not fatal to 50% of a population irrespective of exposure time^34^. This method has decreased in relative popularity over time, due to the large amount of resources required^29^. A few studies have compared these two metrics on a theoretical^40,41^ or empirical^42^ level, but with limited sample sizes.

A third method known as the thermal death time (TDT) was developed concurrently with the critical thermal and incipient lethal temperature methods. This method observes the time elapsed until an endpoint (usually death) occurs at constant temperatures. The role of time in temperature tolerance has been acknowledged since the 1940s, notably by Fry during the development of the incipient lethal temperature procedure^25,34,43^. Recent works have unified thermal tolerance methodologies by considering tolerance in the context of intensity and duration of exposure^40,41,44–46^. Thermal death time studies fall outside of the scope of the present study, as the TDT metric (time) does not directly match the temperature metric of the other methodologies. While mathematical models could resolve this discrepancy^40,44,45^, the present work includes only reported metrics that can be tracked to a primary source.

With the advanced understanding of climate change effects, large collections of thermal limits have been compiled to assess the vulnerability of organisms to warming on a global scale and to answer fundamental questions on the determinants of thermal tolerance^47–52^. Freshwater taxa are not well-represented within these collections, with the exception of amphibians^47,52–54^. Freshwaters support disproportionately high levels of biodiversity and provide vital resources for people^55^. Freshwater ecosystems, including rivers, lake, and wetlands, cover approximately 3% of Earth’s surface but provide habitats for over 50% of all described fish species and a third of all known vertebrates^56,57^. In addition, these systems provide services such as drinking water, water purification, and contribute to climate regulation. Freshwater ecosystems are directly threatened by global warming on a large scale, and locally by thermal pollution from industrial effluents^58–60^. Moreover, climate change may also indirectly increase water temperatures. For example, flow intermittence induced by climatic changes may indirectly result in strongly increased water temperatures^6,61^. Other stressors, such as hydromorphological changes and removal of riparian vegetation can also be associated with increased water temperature^6,62,63^. Evaluating the effects of increasing temperatures on biodiversity requires information on species’ thermal tolerance. Hence, we established the ThermoFresh database focusing on the thermal tolerance of freshwater taxa, which allows for intra- and interspecific comparisons among global freshwater assemblages, assessment of thermal vulnerability under warming scenarios, and prediction of future range shifts.

Thermal tolerance can also change in the presence of other stressors^53,64^, which affect its ecological relevance in a world where many organisms are increasingly subjected to multiple stressors^58,65^. We include thermal tolerance tests in the presence of additional stressors, with information on the type and level of the stressor(s). This facilitates future use of the database in disentangling complex interactions between multiple stressors and consequential effects on organisms in stressful environments.

The database was developed following current recommendations of best practice^66–68^. In comparison to previous studies we aimed to reduce geographic bias, provide multiple records per species (where possible, to allow assessment of intraspecific variation), and report measures of dispersion, to address gaps identified in recent literature^7,54^. We have included thermal tolerance metrics determined from the critical thermal method or the incipient lethal method, including those conducted on the same species within the same study^42,69^, which facilitates comparison of how the metrics are related across taxonomic groups. Our database focuses on freshwater fish and invertebrates, with records collated from previous compilations and expanded with new literature searches, including in widely spoken languages other than English. Including widely spoken non-English languages in ecological literature searches decreases geographical bias, which is critical for extrapolating results across the diverse regions of the earth^70,71^. The current uncertainty induced by geographical bias in physiological data overrides the uncertainty in future climate predictions^72^, and non-English literature can help counteract such bias^73–75^. Additionally, the publication rate of non-English studies on biodiversity is growing in many countries^76^. The relevance of non-English literature has been increasingly recognized^70,71,73^.

## Methods

### Workflow

**Fig. 1:**
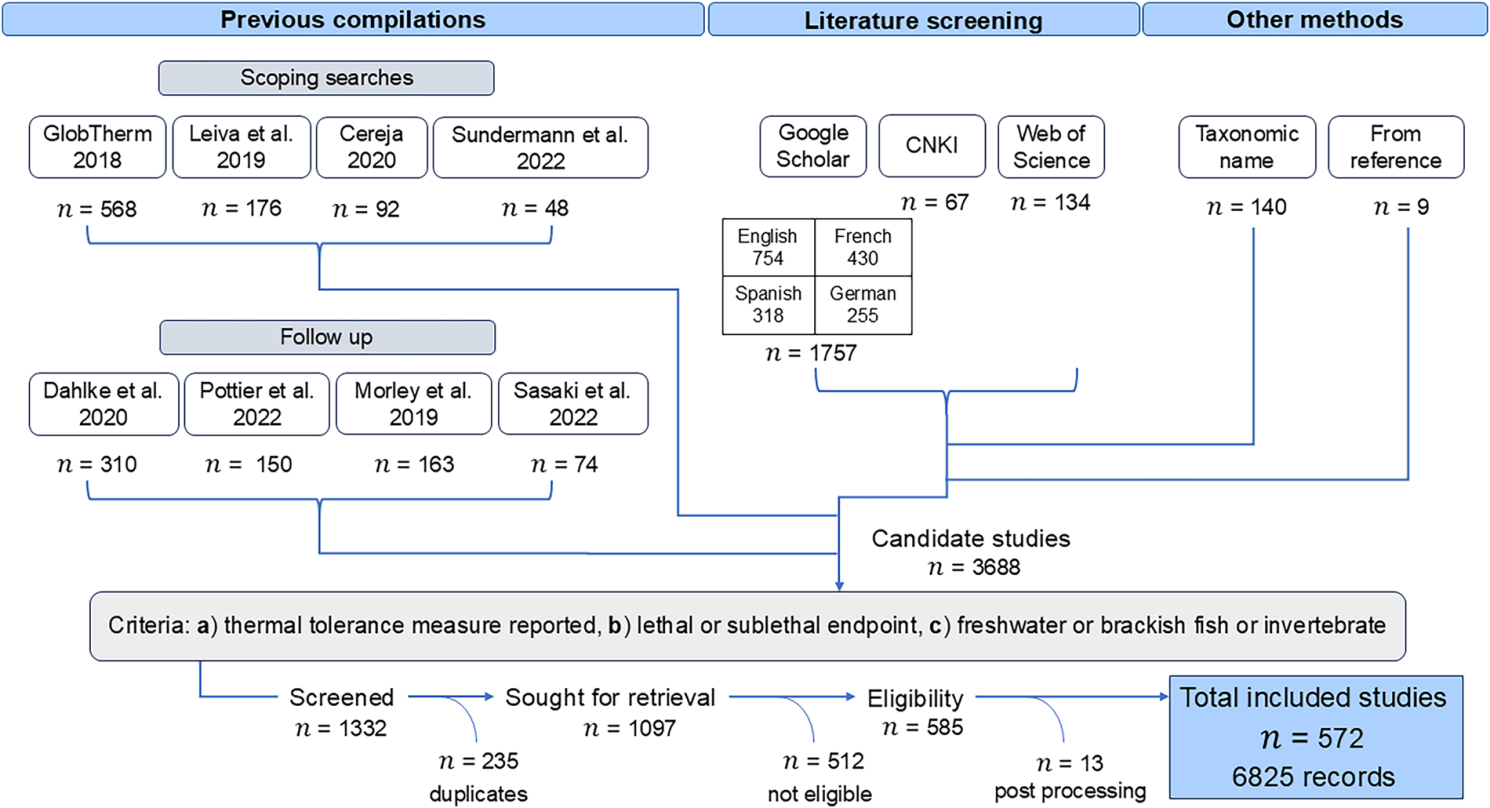
A flow chart describing the workflow to obtain studies included in the database. A total of 572 studies were included culminating in 6825 records of thermal tolerance in total. Nine studies were obtained from references.

The conceptualization of the database started with two previous compilations, the GlobTherm database and a study by Leiva and colleagues^47,52^. The search terms used in these studies were implemented in scoping searches, during which two additional compilations, authored by Cereja^53^ and Sundermann and colleagues^77^, respectively, were found. These four recently published databases were initially selected based on their size, freshwater taxa inclusion, and date of publication. The reasons for the inclusion or exclusion of previous databases are listed in Table 1. To cover gaps in previous publications, we conducted searches in Chinese, German, French, and Spanish up until April 2023, and searches in English until April 2023 in Web of Science as well as from 2019 until April 2023 in Google Scholar. English search terms are listed in Table 2, and all search terms in Supplementary Table 1. During the searches it became apparent that invertebrates were severely underrepresented compared to fish. Therefore, we exerted additional search effort to increase the coverage of invertebrates in the database, using taxonomic names of common invertebrates in the search terms. Throughout this study, several additional compilations^51,78–87^ were discovered, most newly published (Table 1). Of these, four were selected, then harmonized and added to the database following the same procedure as before. The complete workflow is visualized in Fig. 1.

**Table 1:** A list of previously published large collections of thermal tolerance data considered for harmonization in this study, with information on when it was found and the reason for inclusion or exclusion.

Collection	Found	Include	Reason
Bennett *et al*.^47^ (GlobTherm)	Conceptualization	yes	Most species in a single collection to date, includes freshwater taxa
Leiva *et al*.^52^	Conceptualization	yes	Large collection including freshwater taxa
Lutterschmidt & Hutchinson^29^	Scoping searches	no	Age, duplicated in later collections
Beitinger *et al*.^18^	Scoping searches	no	Age, included in GlobTherm
Bates *et al*.^90^	Scoping searches	no	Age, duplication with other collections, common authors with later collections (Morley, Bates, Sunday)
Chown *et al*.^91^	Scoping searches	no	Age, duplication with other collections
Rohr *et al*.^88^	Scoping searches	no	Focus on amphibians
Sasaki & Dam^89^	Scoping searches	no	Only copepods, only freshwater source is duplicate
Cereja^53^	Scoping searches	yes	Aquatic ectotherms, including freshwater taxa
Sundermann *et al*.^77^	Scoping searches	yes	Freshwater invertebrates
Gunderson & Stillmann^85^	References	no	Age
Comte & Olden^84^	References	no	Entirely duplicated in Dahlke *et al*.^81^
Morley *et al*.^51^	References	yes	Large collection of thermal tolerance including freshwater taxa
Hasnain *et al*.^78^	After first search	no	Age, duplication with other collections
Pinsky *et al*.^86^	After first search	no	No freshwater taxa
Barbarossa *et al*.^87^	After first search	no	Source compilations already included
Welch & Jager^79^	After first search	no	Meta-compilation, all source compilations either irrelevant or already included
Bonacina *et al*.^80^	After first search	no	Lack of relevant metrics
Dahlke *et al*.^81^	After first search	yes	Additional papers for freshwater fish
Pottier *et al*.^82^	After first search	yes	Recent collection including freshwater taxa
Sasaki *et al*.^83^	After first search	yes	Recent collection including freshwater taxa

**Table 2:** Search strings for the literature searches conducted in English, along with the targeted organism group and platform within which the search was conducted.

Language	Search string	Group	Search platform	Conducted by
English	“ctmax” AND “freshwater” AND “aquatic” AND “critical thermal maximum”	Fish, invertebrates	Google Scholar	HSB
English	“lt50” AND “freshwater” AND “aquatic” AND “upper thermal tolerance”	Fish, invertebrates	Google Scholar	HSB
English	“freshwater” AND (“critical thermal maximum” OR “upper thermal tolerance” OR “lower thermal tolerance” OR “thermal tolerance breadth” OR “heat tolerance” OR “upper lethal temperature limit” OR “thermal tolerance window” OR “species temperature tolerance” OR “ctmax”)	Fish, invertebrates	Web of Science	HSB
English^a^	“*taxonomic name*” AND “thermal tolerance” OR “heat tolerance” OR “ctmax” OR “incipient lethal temperature” OR “critical thermal maximum” OR “thermal limit”	Invertebrates	Google Scholar	HSB

Search terms for all languages can be found in Supplementary Table 1.

^a^The search terms were iterated across a list of taxonomic names for this search, with the respective taxonomic name serving as the first term in the search string.

### Inclusion criteria

The database includes studies that fulfill three primary criteria: 1) report at least one thermal tolerance metric for an organism, 2) have mortality or a sublethal indication of imminent mortality (such as loss of equilibrium, loss of righting response, or onset of spasms) as an endpoint, and 3) test organisms are fish or invertebrates residing in fresh or brackish water habitats for at least part of their life cycle (Fig. 1). Studies that contained incomprehensible or missing methodology for thermal tolerance tests were excluded; minor methodological inconsistencies were noted during data extraction. The thermal tolerance metrics considered for inclusion needed to represent the result of either a dynamic or static assay, and either contain enough methodological information to deduce which kind of assay was conducted or cite the recognized metric name (CTmax, CTmin, LTmax, LTmin, LT50, IULT, ILLT) in accordance with literature describing methodology (notably key papers such as Cowles and Bogert 1944, Becker and Genoway 1979, Lutterschmidt and Hutchinson 1997, Beitinger and Bennett 2000)^18,19,23,29^.

### Previous databases

We initially selected four recently published databases on thermal tolerance that include freshwater organisms and contain variables relevant to the test organisms and the test metrics. These were identified via scoping searches on thermal tolerance literature. The GlobTherm database^47^ contains thermal tolerance records for the largest number of species in one compilation to date, the compilation of Leiva and colleagues^52^ includes a high proportion of freshwater invertebrate taxa, the compilation by Cereja^53^ includes only aquatic species (therefore more freshwater taxa) and notes additional stressors^53^, and the dataset by Sundermann and colleagues^77^ focuses exclusively on freshwater invertebrates. Other large datasets either contained fewer relevant variables due to the nature of the research questions they were collected for^51,88^, fewer freshwater taxa (especially invertebrates)^54,89^, or a high duplication of studies with the selected databases^90,91^. We excluded collections published before 2018, given the high duplication with more recent literature (Table 1).

We attempted to harmonize the selected databases. However, previous databases either only reported one value per taxon, even when multiple values were reported in constituent studies, mislabelled thermal tolerance metrics (i.e. labelling all thermal tolerance values as CTmax, though some were other metrics), or lacked key information related to the method, location, or test organism. Thus all selected databases were filtered for relevant freshwater or brackish taxa, all existing data for these taxa combined, and each corresponding source reference examined to cross-check existing data, correct errors, and fill in missing variables. This included extracting additional data from the main text, tables, figures, and supplementary information of source studies. Additional records were added when present in the original source. When data was only presented in figures, it was extracted using Plot Digitizer^92^. Collected variables are summarized in Table 3, and the extraction protocol with descriptions of all variables in detail is available in the “methods” folder on GitHub (https://github.com/hsbayat/ThermoFresh/tree/main/methods; see also Code Availability section).

**Table 3:** An overview of the variables included for records in the database.

Variable type	Variables	Variables queried
Test organism	Taxon, common name, life stage, sex, body mass, body length, group, habitat, origin	Habitat classification queried from WORMS
Taxonomy	Domain, kingdom, phylum, class, order, family, genus, species, subspecies	Queried from the Open Tree of Life
Thermal tolerance and method	Tolerance value, metric, sample size, error, error measure, acclimation time, acclimation temperature, ramp rate, test time, endpoint, aeration, pH, salinity, oxygen	
Environmental information	Environmental temperature at collection, environmental temperature maximum/minimum, season, season type, climate region, continent, country, elevation	Köppen-Geiger climate region, elevation, continents, and countries queried from R package rnaturalearth using coordinates
Sampling information	collection year, collection dates, latitude and longitude of collection location	
Stressor information	Stressor type, stressor level, stressor units	
Reference information	Reference code, full reference citation, doi, journal, publication year	
Notes	General notes, metric note, taxon name note, endpoint note, stressor note, body size note, origin note	
Other	Test id, study id, taxon id, location id, ott id	OTT ID queried from the Open Tree of Life using the taxon name

Four additional large collections were identified during and after the search and data extraction process (Table 1). The compilation by Dahlke and colleagues^81^ focuses on fish, including many sources from the grey literature, and all papers compiled by Comte & Olden 2017^84^. Collections by Morley and colleagues^51^, Pottier and colleagues^82^, and Sasaki and colleagues^83^ all include invertebrates as well as fish. These were filtered for freshwater taxa and screened for duplicate sources; then data was extracted from each original source as described above.

### Literature searches

We complemented the selection of records from existing compilations with new literature searches. A general literature search was conducted in Google Scholar in English for the time period from January 1, 2019 until April 26, 2023, to cover studies that appeared after the previous databases were compiled, with search terms targeting both the critical thermal and incipient lethal methodology (Table 2). Far more studies were obtained using search terms targeting the critical thermal method than those targeting the incipient lethal temperature. We concentrated search efforts on upper thermal tolerance, but also extracted lower thermal tolerance where it was reported. We also conducted a search in Web of Science from the earliest index date until April 26, 2023. Pilot searches were conducted in all languages to fine-tune the search terms. Final searches in French, German, and Spanish were conducted in Google Scholar, and the search in Chinese was conducted in the Chinese National Knowledge Infrastructure (CNKI), for the earliest indexed studies up until April 26, 2023 (Supplementary Table 1). The pilot search and search term refinement for the Spanish search were performed in the Scientific Electronic Library Online (SciElo) and Google Scholar. More relevant publications were indexed in Google Scholar, so the final search was conducted there. Pilot searches in Italian and Norwegian did not result in any relevant publications. One publication in Danish came up as part of the English search and was included. Papers in other languages were excluded.

Fish made up the majority of freshwater taxa in existing databases, while invertebrates were vastly underrepresented^47^. The GlobTherm database containing the most taxa (over 2000) includes only 8 species of freshwater insects^47^, a four-fold underrepresentation according to current estimates of the total species on earth^93,94^. Therefore we exerted additional effort to find papers focused on the thermal tolerance of freshwater invertebrates. We deemed this extra effort unnecessary for fish, since freshwater fish were already well-represented in existing collections, with the large compilation by Dahlke and colleagues focusing on fish entirely. Freshwater invertebrate species outnumber freshwater fish at least seven to one; freshwater insect species alone outnumber freshwater fish species five to one^95^, yet fish species outnumber invertebrate species in all existing collections covering both groups.

Scoping searches confirmed that key papers focused on invertebrates were partly missed in searches with general search terms. We then modified the general English search terms by adding the order, family, or genus name of freshwater invertebrates obtained from a list of freshwater invertebrates sampled in Germany over the last 12 years, spanning habitats from near-natural to highly degraded conditions^96,97^. The orders and families from this list are distributed globally^56^, and contain many of the most studied taxa. Papers resulting from these searches were not limited to one geographic region. Nonetheless we expanded the search to include global invertebrate families from Africa, Asia, Europe, North America, Oceania, and South America^98,99^. A full list of all taxonomic names used in the search is found in the “methods” folder on GitHub (see Code Availability section). The expanded search resulted in 6 additional eligible studies, compared to 67 resulting from the initial list.

Google Scholar search results were saved as a file using Publish or Perish v8 software^100^. Search results were screened first for relevance by title and abstract, then for duplicates with the studies already included from previous databases. Some studies were eliminated during the data extraction phase because inclusion criteria were not met, in these cases the reason for elimination was noted.

### Data extraction and processing

Data was extracted according to a standardized protocol. Following data extraction, a subset of the data was cross-checked against the source references by three authors. Data was also examined for outliers and unreasonable values indicative of errors (for instance, a weight of 2 kilograms for invertebrate larvae was reported, which was caused by a missed decimal point) were corrected. Spelling errors were also checked and corrected. The endpoint was scored variably by different authors, though it was often essentially the same. These were unified into simpler categories and details relegated to the notes. All categorical variables were examined and corrected for consistency; all variables and their categories are described in the full metadata on GitHub (https://github.com/hsbayat/ThermoFresh/tree/main/data/metadata). Once datasets from all authors were compiled and errors checked, coordinates were used to query the Köppen-Geiger climate region^101^, elevation, continent, and country of each sampling location. Missing values, primarily due to coastal proximity, were entered manually. Full taxonomic information for each taxon was queried from the Open Tree of Life^102^, which collates taxonomic information from multiple sources. To do this, Open Tree Taxonomy (OTT) identifiers were queried for all taxa names in the database, then taxonomic classifications were added for each taxon using the OTT identifier. All screening, harmonization, data processing, querying, and plotting were conducted in R v4.3.3^103^ with the following packages: revtools v0.4.1^104^, tidyverse v2.0.0^105^, data.table v1.14.10^106^, rotl v3.1.0^107^, taxize v0.9.100^108^, sf v1.0-15^109^, elevatr v0.99.0^110^, geodata v0.5-9^111^, terra v1.7-65^112^, fs v1.6.3^113^ rnaturalearth v1.0.1^114^, viridis v0.6.5^115^, patchwork v1.2.0^116^, ggridges v0.5.6^117^.

## Data Records

ThermoFresh is split into four tables: thermal tolerance test information, reference information, taxonomic information, and location information. A combined table with all data, and code to combine them, is available on GitHub (https://github.com/hsbayat/ThermoFresh). Code and data are also available at Zenodo^118^. Full metadata is available in the “metadata” subfolder in the GitHub repository. All data files in the repository are saved as comma-separated values files (.csv). Reference information, including DOI where available, is provided in the reference table (thermtol_reference_final_ch.csv) as well as for each record in the combined table (thermtol_comb_final.csv). Reference information in the original language is also provided.

**Fig. 2:**
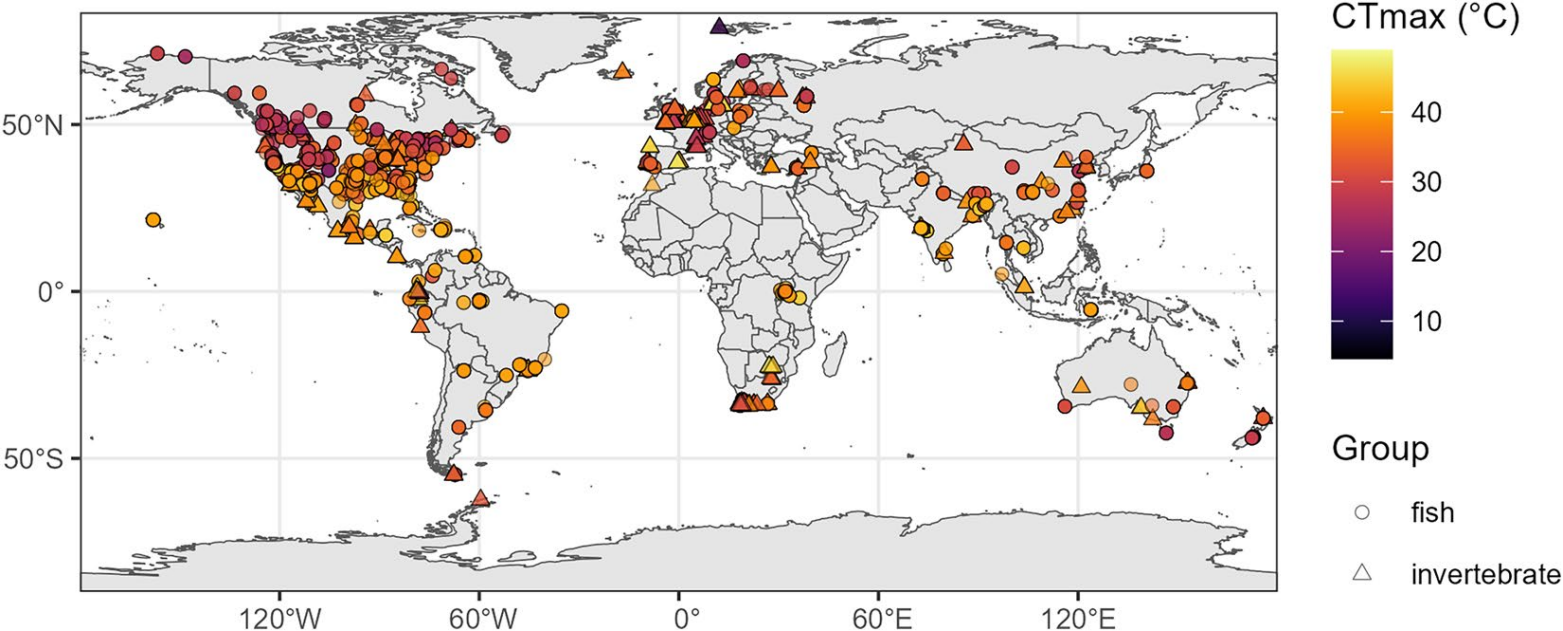
A map of the critical thermal maxima (CTmax) values in the database; the color ramp indicates the value in degrees Celsius.

**Fig. 3:**
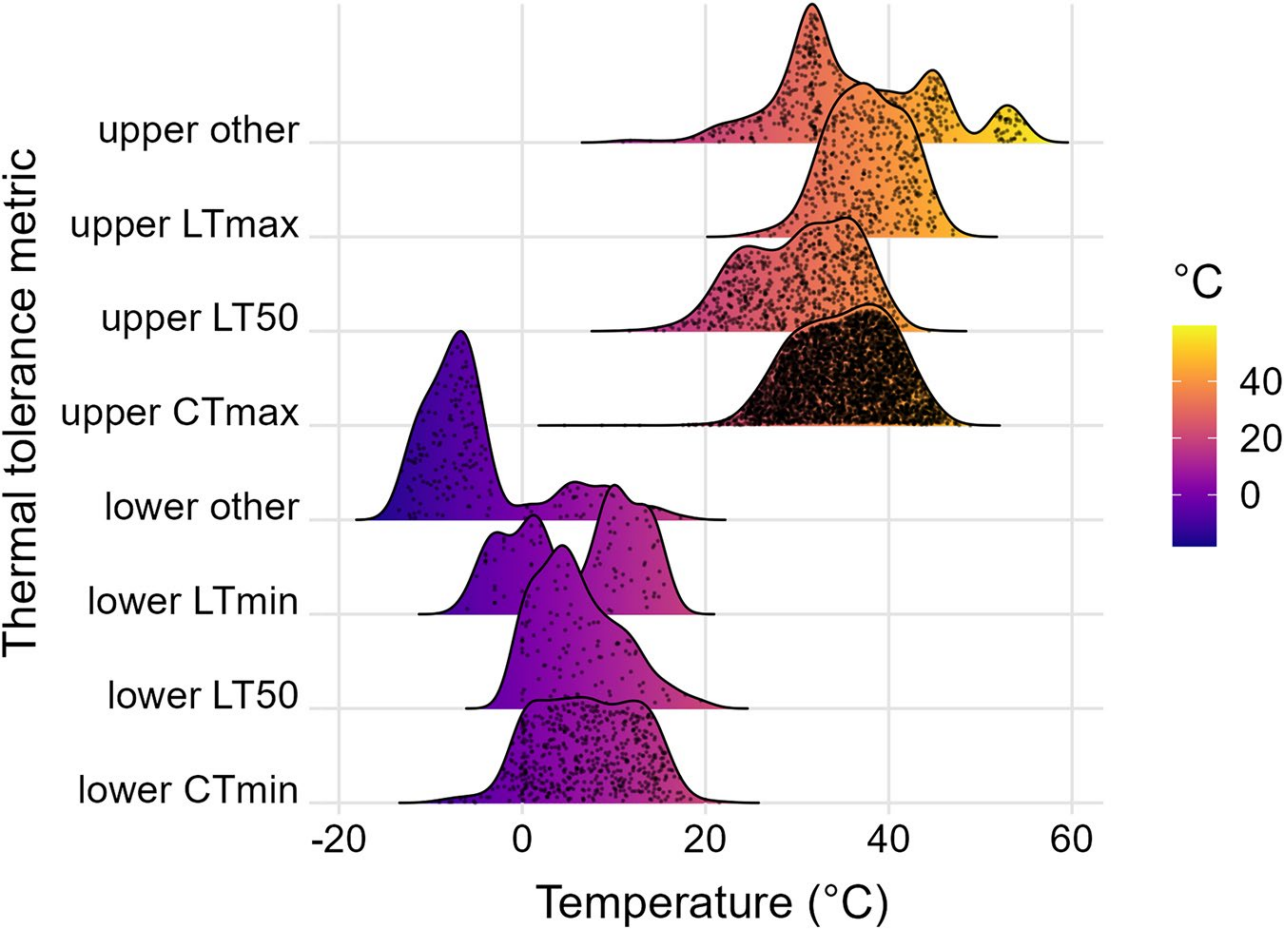
A comparison of the density distributions of thermal tolerance values in the database for each metric. The points underneath each curve represent the sample size; with n = 460, 409, 692, 4362, 172, 83, 94, and 553 from top to bottom (upper other, upper LTmax, upper LT50, upper CTmax, lower other, lower LTmin, lower LT50, and lower CTmin, respectively). The fill color indicates the tolerance value in degrees Celsius.

**Fig. 4:**
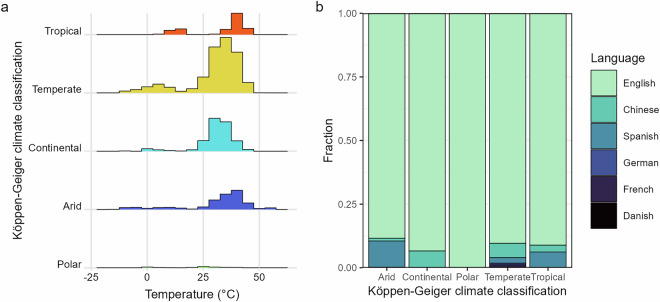
A comparison of all tolerance values by Köppen-Geiger climate region; (**a**) the distributions of tolerance values within each group, scaled by the sample size, and (**b**) the fraction of values within each group by source language.

**Fig. 5:**
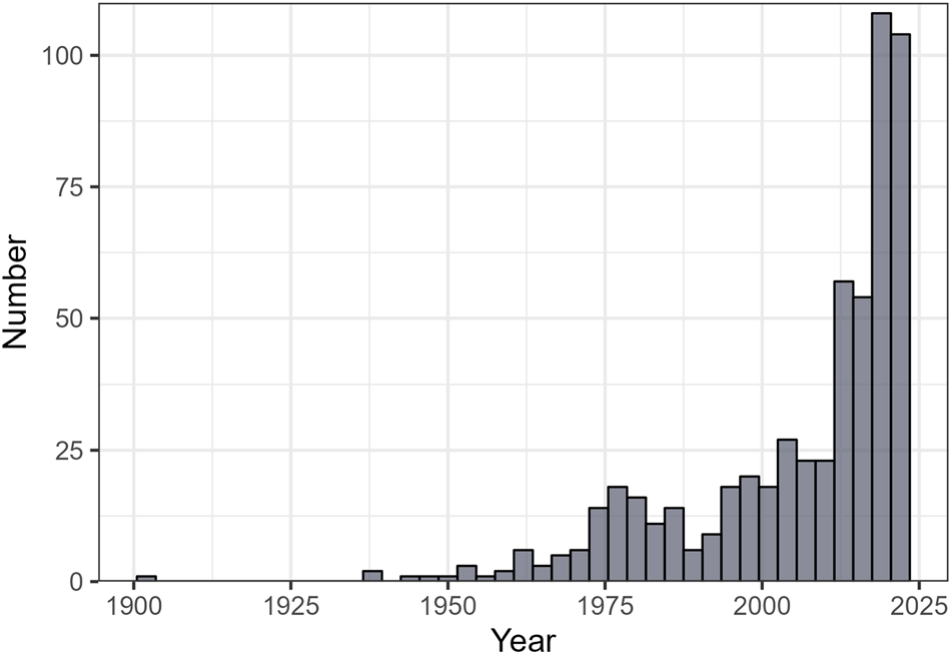
Histogram of the number of studies per publication year included in the database. Publication date spans from 1900 until 2023.

The database includes 6825 records for a total of 931 taxa, 470 invertebrates and 461 fish, from 1082 locations worldwide (Fig. 2). The database contains primarily organisms identified to the species level, with 86 taxa at the genus level and 8 above the genus level. Of the 931 total taxa, 666 reside solely in freshwater, 73 only in brackish waters, and 192 in both. A total of 505 tests recorded thermal tolerance of species in the presence of an additional stressor (e.g., pollutants, pathogen, reduced oxygen, flow velocity, salinity). The inclusion of variables like body size, life stage, coordinates, and environmental conditions allows for a wide range of hypotheses to be tested with this data. The detailed information on test metrics and methodology allows for metrics to be compared; Fig. 3 illustrates the density distributions for each metric. Records can be filtered according to desired features, e.g. a high or low acclimation temperature. Thermal tolerances from different climates can also be compared (Fig. 4a). Non-English languages account for 620 records, with 323 Chinese, 235 Spanish, 34 French, 21 German, and 7 Danish records. Non-English studies contributed most to records tested in arid climates, followed by temperate, tropical, and continental (Fig. 4b). The database features records from 1900 until 2023, which presents the opportunity for comparisons across time, though these may be limited by the scarcity of early data (Fig. 5).

## Technical Validation

Data was extracted from the original literature sources for all studies resulting from literature searches; a note was made when data was found in supplementary information or extracted with Plot Digitizer^92^. Data was cross-checked with the original study for each record obtained from previous databases. A manual double-check of data entry with reference to the original source was done for 20.8% of all records by at least one additional person. Additionally the distributions of all numerical variables was visualized in R^103^, and outliers were manually checked. Outliers were defined as values exceeding 1.5 times the interquartile range above and below the first and third quartiles. Typos in taxon names were corrected, and outdated species names changed to currently accepted names (as of July 2024 in the Open Tree of Life Taxonomy). The names as referred to in the original source are in the notes column, where names were changed. The OTT identifier, which is included as a variable, allows for names to be updated more easily should they change in the future.

All records include a taxon name at the genus or species level, the origin of the test taxon, a thermal tolerance measure, the metric (indicating which methodology was used), the endpoint, the habitat, the location of sampling (or laboratory location for non-wild organisms), variables relating to the location (continent, country, elevation), and reference information (including publication year and language). Over 90% of records include sample size, acclimation information, and the life stage of the organism at the time of the test. Error measures are included for 65% of records; the type of error measure is also noted in all cases where error is included. This information, or lack thereof, can be used to filter the data as needed to investigate various research questions. The variables describing test methods (ramp, duration) allow for integration of the tolerance metrics into mathematical models of the tolerance landscape framework^41,44,45^. These contextualize tolerance with respect to intensity and duration of thermal stress, and enable extrapolation to field conditions^119^.

The multi-faceted search strategy involving harmonization of previous literature, additional literature searches in multiple languages across three search platforms (Google Scholar, China National Knowledge Infrastructure, and Web of Science), produced a comparably comprehensive compilation of thermal tolerance for freshwater invertebrates and fish. The concentrated search effort added 299 invertebrate taxa, nearly double the 171 taxa obtained from existing databases. Of 470 freshwater or brackish invertebrates, 322 are insects, representing 0.4% of the currently estimated total number of species^93,95^. While this may seem low, it is several hundred to thousand-fold more than previous work focused on all taxa^47,52,53^. The 461 freshwater fish species in the database represent 3% of the currently estimated number of species^95^.

While we attempted to counteract geographic bias in our approach, Europe and North America represent 65.6% of total records in the database though they make up 23.7% of Earth’s land area. With only English studies included, they would make up 70% of the records. While non-English languages contribute to lessening data gaps (Fig. 2), considerable work remains to fill large gaps in the geographic distribution of data.

When it comes to data gaps, multiple factors, including political ones, are at play. Data may simply be absent from certain areas, due to a lack of access, interest, or resources. In these areas more research and resources are needed to increase data coverage. However, data may also be locked in older studies which are much more difficult to access than more recently published work. For instance, thermal tolerance papers concerned with the deleterious effects of thermal effluents from power plants were prevalent in the time period from 1960 to 1980. While scans of these are common in US archives that are indexed by Google Scholar, the archives of other countries, which almost certainly also had research programs on the topic^120^, are difficult or impossible to access for non-native researchers. Screening literature in non-English languages requires resources, skill, and collaboration, but can play a key role in countering bias and harnessing information from locations that are least covered.

Given the continual increase in volume of literature on thermal tolerance (Fig. 5), on par with scientific research as a whole^121^, we expect our work to benefit from an update in the future. The search terms provided can be used to conduct equivalent searches and expand the database. Advances in automated data extraction^122–125^ foretell the automation of data synthesis research, which can enhance efforts to update synthetic work amidst ever-increasing output. So far, full automation of data extraction has been conducted for only few ecological variables^122^ or in medical research^123–125^. Medical research enforces stringent reporting guidelines for individual studies, which assist data synthesis and allow automated methods to operate effectively. Reporting guidelines have also been introduced for terrestrial respirometry^126^, climate change genomics^127^, and landscape ecology^128^, but have yet to be developed for thermal tolerance studies. A well-curated database such as ours can serve as a reference, benchmark, and training tool in future efforts to continuously synthesize new information on freshwater thermal tolerance.

## Supplementary information

Supplementary Table 1

## Data Availability

The code to process and aggregate the data and reproduce all figures is available on GitHub: https://github.com/hsbayat/ThermoFresh. The “methods” folder contains the data extraction protocol and additional methodological information.

## References

[1] Spallanzani. *Opuscules de Physique, Animale et Vegetale*. (Chez Barthelemi Chirol, Geneva, 1777).

[2] Plateau, F. *Recherches physico-chimiques sur les articules aquatiques*. (F. Hayez, Imprimeur de l’Acadamie Royale de Belgique, Brussels, 1870).

[3] Davenport, C. B. & Castle, W. E. Acclimatization of Organisms to High Temperatures 1) (1895).

[4] Davy, J. Some Observations on the Ova of the Salmon, in relation to the distribution of Species (1855).

[5] Hoppe-Seyler, F. Ueber die obere Temperaturgrenze des Lebens. *Arch. Für Gesammte Physiol. Menschen Thiere* **11**, 113–121 (1875).

[6] Desforges, J. E. *et al*. The ecological relevance of critical thermal maxima methodology for fishes. *J. Fish Biol.* **102**, 1000–1016 (2023). 36880500 10.1111/jfb.15368

[7] Herrando-Pérez, S., Vieites, D. R. & Araújo, M. B. Novel physiological data needed for progress in global change ecology. *Basic Appl. Ecol.* **67**, 32–47 (2023).

[8] Chown, S. L. Macrophysiology for decision-making. *J. Zool.* **319**, 1–22 (2023).

[9] Jutfelt, F. *et al*. Oxygen-and capacity-limited thermal tolerance: blurring ecology and physiology, 10.1242/jeb.169615 (2018). 10.1242/jeb.169615 29321291

[10] Verberk, W. C. E. P. *et al*. Does oxygen limit thermal tolerance in arthropods? A critical review of current evidence. *Comp. Biochem. Physiol. -Part Mol. Integr. Physiol.* **192**, 64–78 (2016). 10.1016/j.cbpa.2015.10.020 PMC4717866 26506130

[11] Camacho, A. *et al*. Does heat tolerance actually predict animals’ geographic thermal limits? *Sci. Total Environ.* **917**, 170165 (2024). 38242475 10.1016/j.scitotenv.2024.170165

[12] Gouveia, S. F. *et al*. Climatic niche at physiological and macroecological scales: the thermal tolerance–geographical range interface and niche dimensionality. *Glob. Ecol. Biogeogr.* **23**, 446–456 (2014).

[13] Gutiérrez-Pesquera, L. M. *et al*. Testing the climate variability hypothesis in thermal tolerance limits of tropical and temperate tadpoles. *J. Biogeogr.* **43**, 1166–1178 (2016).

[14] Comte, L. & Olden, J. D. Evolutionary and environmental determinants of freshwater fish thermal tolerance and plasticity. *Glob. Change Biol.* **23**, 728–736 (2017). 10.1111/gcb.13427 27406402

[15] Perez, T. M. & Feeley, K. J. Weak phylogenetic and climatic signals in plant heat tolerance. *J. Biogeogr.* **48**, 91–100 (2021).

[16] Kellermann, V. *et al*. Upper thermal limits of Drosophila are linked to species distributions and strongly constrained phylogenetically. **109**, 16228–16233 (2012). 10.1073/pnas.1207553109 PMC3479592 22988106

[17] Bodensteiner, B. L. *et al*. Thermal adaptation revisited: How conserved are thermal traits of reptiles and amphibians? *J. Exp. Zool. Part Ecol. Integr. Physiol.* **335**, 173–194 (2021). 10.1002/jez.2414 32970931

[18] Beitinger, T. L., Bennett, W. A. & Mccauley, R. W. Temperature tolerances of North American freshwater fishes exposed to dynamic changes in temperature. *Environ. Biol. Fishes* **58**, 237–275 (2000).

[19] Becker, C. D. & Genoway, R. G. Evaluation of the critical thermal maximum for determining thermal tolerance of freshwater fish. *Env Biol Fish* **4**, 245–256 (1979).

[20] Lutterschmidt, W. I. & Hutchison, V. H. The critical thermal maximum: data to support the onset of spasms as the definitive end point. *Can. J. Zool.* **75**, 1553–1560 (1997).

[21] Golovanov, V. K. Influence of various factors on upper lethal temperature (review). *Inland Water Biol.* **5**, 105–112 (2012).

[22] Bates, A. E. & Morley, S. A. Interpreting empirical estimates of experimentally derived physiological and biological thermal limits in ectotherms 1. *Can. J. Zool.* **98**, 237–244 (2019).

[23] Cowles, R. B. & Bogert, C. M. A preliminary study of the thermal requirements of desert reptiles. *Bull. AMNH* **83** (1944).

[24] Huntsman, A. G. & Sparks, M. I. No. 6: Limiting Factors For Marine Animals.: 3. Relative Resistance To High Temperatures. *Contrib. Can. Biol. Fish.* **2**, 95–114 (1924).

[25] Doudoroff, P. The resistance and acclimatization of marine fishes to temperature changes. i. Experiments with Girella nigricans (ayres). *Biol. Bull.* **83**, 219–244 (1942).

[26] Rezende, E. L. & Bozinovic, F. Thermal performance across levels of biological organization, 10.1098/rstb.2018.0549 (2019). 10.1098/rstb.2018.0549 PMC6606466 31203764

[27] Clusella-Trullas, S., Garcia, R. A., Terblanche, J. S. & Hoffmann, A. A. How useful are thermal vulnerability indices? **36** (2021). 10.1016/j.tree.2021.07.001 34384645

[28] Huey, R. B. & Stevenson, R. D. Integrating Thermal Physiology and Ecology of Ectotherms: A Discussion of Approaches. *Am. Zool.* **19**, 357–366 (1979).

[29] Lutterschmidt, W. I. & Hutchison, V. H. The critical thermal maximum: history and critique. *Can. J. Zool.* **75**, 1561–1574 (1997).

[30] Garcia-Robledo, C., Kuprewicz, E. K., Dierick, D., Hurley, S. & Langevin, A. The affordable laboratory of climate change: devices to estimate ectotherm vital rates under projected global warming. *Ecosphere* **11**, e03083 (2020).

[31] Gilbert, M. J. H. *et al*. The thermal limits of cardiorespiratory performance in anadromous Arctic char (Salvelinus alpinus): a field-based investigation using a remote mobile laboratory. *Conserv. Physiol.* **8**, coaa036 (2020). 32346481 10.1093/conphys/coaa036 PMC7176916

[32] Chatterjee, N., Pal, A. K., Manush, S. M., Das, T. & Mukherjee, S. C. Thermal tolerance and oxygen consumption of Labeo rohita and Cyprinus carpio early fingerlings acclimated to three different temperatures. *J. Therm. Biol.* **29**, 265–270 (2004).

[33] Fry, F. E. J. Effects of the environment on animal activity. *Publ Fish Res Lab* **55**, 1–62 (1947).

[34] Fry, F. E. J., Hart, J. S. & Walker, K. F. Lethal temperature relations for a sample of young speckled trout. *Univ. Tor. Stud. Biol. Ser.* **54**, 9–35 (1946).

[35] Fry, F. E. J., Brett, J. R. & Clawson, G. H. Lethal limits of temperature for young goldfish. *Rev Can Biol* **1**, 50–56 (1942).

[36] Hathaway, E. S. *Quantitative Study of the Changes Produced by Acclimatization in the Tolerance of High Temperatures by Fishes and Amphibians*. vol. 1030 (US Government Printing Office, 1927).

[37] Bliss, C. I. The Calculation of the Dosage-Mortality Curve. *Ann. Appl. Biol.* **22**, 134–167 (1935).

[38] Bliss, C. I. & Stevens, W. L. The Calculation of the Time-Mortality Curve. *Ann. Appl. Biol.* **24**, 815–852 (1937).

[39] Brett, J. R. Some lethal temperature relations of Algonquin Park fishes. (1944).

[40] Cooper, B. S., Williams, B. H. & Angilletta, M. J. Unifying indices of heat tolerance in ectotherms. *J. Therm. Biol.* **33**, 320–323 (2008).

[41] Jørgensen, L. B., Malte, H., Ørsted, M., Klahn, N. A. & Overgaard, J. A unifying model to estimate thermal tolerance limits in ectotherms across static, dynamic and fluctuating exposures to thermal stress. *Sci. Rep.* **11**, 1–14 (2021). *2021 111*. 34145337 10.1038/s41598-021-92004-6 PMC8213714

[42] Dallas, H. F. & Ketley, Z. A. Upper thermal limits of aquatic macroinvertebrates: Comparing critical thermal maxima with 96-LT50 values. *J. Therm. Biol.* **36**, 322–327 (2011).

[43] Doudoroff, P. The resistance and acclimatization of marine fishes to temperature changes. ii. Experiments with Fundulus and Atherinops. *Biol. Bull.* **88**, 194–206 (1945).

[44] Rezende, E. L., Castañeda, L. E. & Santos, M. Tolerance landscapes in thermal ecology. *Funct. Ecol.* **28**, 799–809 (2014).

[45] Ørsted, M., Jørgensen, L. B. & Overgaard, J. Finding the right thermal limit: a framework to reconcile ecological, physiological and methodological aspects of CTmax in ectotherms. *J. Exp. Biol.* **225**, jeb244514 (2022). 36189693 10.1242/jeb.244514

[46] Semsar-kazerouni, M. & Verberk, W. C. E. P. It’s about time: Linkages between heat tolerance, thermal acclimation and metabolic rate at different temporal scales in the freshwater amphipod *Gammarus fossarum* Koch, 1836. *J. Therm. Biol.* **75**, 31–37 (2018). 30017049 10.1016/j.jtherbio.2018.04.016

[47] Bennett, J. M. Data Descriptor: GlobTherm, a global database on thermal tolerances for aquatic and terrestrial organisms Background & Summary. 10.1038/sdata.2018.22 (2018). 10.1038/sdata.2018.22 PMC5848787 29533392

[48] Bennett, J. M. *et al*. The evolution of critical thermal limits of life on Earth. *Nat. Commun.* **12**, 1198 (2021). 33608528 10.1038/s41467-021-21263-8 PMC7895938

[49] Sunday, J. M., Bates, A. E. & Dulvy, N. K. Thermal tolerance and the global redistribution of animals. *Nat. Clim. Change 2012* **29**(2), 686–690 (2012).

[50] Comte, L., Murienne, J. & Grenouillet, G. Species traits and phylogenetic conservatism of climate-induced range shifts in stream fishes. *Nat. Commun.* **5**, 1–9 (2014). 10.1038/ncomms6053 PMC5898465 25248802

[51] Morley, S. A., Peck, L. S., Sunday, J. M., Heiser, S. & Bates, A. E. Physiological acclimation and persistence of ectothermic species under extreme heat events. *Glob. Ecol. Biogeogr.* **28**, 1018–1037 (2019).

[52] Leiva, F. P., Calosi, P. & Verberk, W. C. E. P. Scaling of thermal tolerance with body mass and genome size in ectotherms: a comparison between water-and air-breathers. *Philos. Trans. R. Soc. B* **374** (2019). 10.1098/rstb.2019.0035 PMC6606457 31203753

[53] Cereja, R. Critical thermal maxima in aquatic ectotherms. *Ecol. Indic*. **119** (2020).

[54] Pottier, P. *et al*. A comprehensive database of amphibian heat tolerance. *Sci. Data* **9**, 600 (2022). 36195601 10.1038/s41597-022-01704-9 PMC9532409

[55] Lynch, A. J. *et al*. People need freshwater biodiversity. *WIREs Water* **10**, e1633 (2023).

[56] *Freshwater Animal Diversity Assessment*, 10.1007/978-1-4020-8259-7 (Springer Netherlands, Dordrecht, 2008).

[57] Carrete Vega, G. & Wiens, J. J. Why are there so few fish in the sea? *Proc. R. Soc. B Biol. Sci.* **279**, 2323–2329 (2012). 10.1098/rspb.2012.0075 PMC3350687 22319126

[58] Jackson, M. C., Loewen, C. J. G., Vinebrooke, R. D. & Chimimba, C. T. Net effects of multiple stressors in freshwater ecosystems: a meta-analysis. 10.1111/gcb.13028 26149723

[59] Coutant, C. C. & Brook, A. J. Biological aspects of thermal pollution I. Entrainment and discharge canal effects∗. *C R C Crit. Rev. Environ. Control* **1**, 341–381 (1970).

[60] Coutant, C. C. & Goodyear, C. P. Thermal Effects. *J. Water Pollut. Control Fed.* **44**, 1250–1294 (1972). 4556360

[61] Chiu, M.-C., Leigh, C., Mazor, R., Cid, N. & Resh, V. Chapter 5.1 - Anthropogenic Threats to Intermittent Rivers and Ephemeral Streams. in *Intermittent Rivers and Ephemeral Streams* (eds. Datry, T., Bonada, N. & Boulton, A.) 433–454, 10.1016/B978-0-12-803835-2.00017-6 (Academic Press, 2017).

[62] Garner, G., Malcolm, I. A., Sadler, J. P. & Hannah, D. M. The role of riparian vegetation density, channel orientation and water velocity in determining river temperature dynamics. *J. Hydrol.* **553**, 471–485 (2017).

[63] Johnson, R. K. & Almlöf, K. Adapting boreal streams to climate change: effects of riparian vegetation on water temperature and biological assemblages. *Freshw. Sci.* **35**, 984–997 (2016).

[64] Cuenca Cambronero, M., Beasley, J., Kissane, S. & Orsini, L. Evolution of thermal tolerance in multifarious environments. *Mol. Ecol.* **27**, 4529–4541 (2018). 30298601 10.1111/mec.14890

[65] Spence, A. R. & Tingley, M. W. The challenge of novel abiotic conditions for species undergoing climate-induced range shifts. *Ecography* **43**, 1571–1590 (2020).

[66] Schwanz, L. E. *et al*. Best practices for building and curating databases for comparative analyses. *J. Exp. Biol.* **225**, jeb243295 (2022). 35258608 10.1242/jeb.243295

[67] Mengist, W., Soromessa, T. & Legese, G. Method for conducting systematic literature review and meta-analysis for environmental science research. *MethodsX* **7** (2020). 10.1016/j.mex.2019.100777 PMC6974768 31993339

[68] O’dea, R. E. *et al*. Preferred reporting items for systematic reviews and meta-analyses in ecology and evolutionary biology: a PRISMA extension. *Biol. Rev*. 0–000, 10.1111/brv.12721 (2021). 10.1111/brv.12721 PMC8518748 33960637

[69] Leggott, M. & Pritchard, G. Thermal preference and activity thresholds in populations of Argia vivida (Odonata: Coenagrionidae) from habitats with different thermal regimes. *Hydrobiologia* **140**, 85–92 (1986).

[70] Amano, T. Tapping into non-English-language science for the conservation of global biodiversity, 10.1371/journal.pbio.3001296 (2021). 10.1371/journal.pbio.3001296 PMC8496809 34618803

[71] Amano, T. *et al*. The role of non-English-language science in informing national biodiversity assessments. *Nat. Sustain.* **6**, 845–854 (2023).

[72] White, C. R. *et al*. Geographical bias in physiological data limits predictions of global change impacts. *Funct. Ecol.* **35**, 1572–1578 (2021).

[73] Konno, K. *et al*. Ignoring non-English-language studies may bias ecological meta-analyses. *Ecol. Evol.* **10**, 6373–6384 (2020). 32724519 10.1002/ece3.6368 PMC7381574

[74] Amano, T., González-Varo, J. P. & Sutherland, W. J. Languages Are Still a Major Barrier to Global Science. *PLOS Biol.* **14**, e2000933 (2016). 28033326 10.1371/journal.pbio.2000933 PMC5199034

[75] Nuñez, M. A. & Amano, T. Monolingual searches can limit and bias results in global literature reviews. *Nat. Ecol. Evol.* **5**, 264–264 (2021). 33398107 10.1038/s41559-020-01369-w

[76] Chowdhury, S. *et al*. Growth of non-English-language literature on biodiversity conservation. *Conserv. Biol.* **36**, e13883 (2022). 34981574 10.1111/cobi.13883 PMC9539909

[77] Sundermann, A., Müller, A. & Halle, M. A new index of a water temperature equivalent for summer respiration conditions of benthic invertebrates in rivers as a bio-indicator of global climate change. *Limnologica* **95** (2022).

[78] Hasnain, S. S., Shuter, B. J. & Minns, C. K. Phylogeny influences the relationships linking key ecological thermal metrics for North American freshwater fish species. *Can. J. Fish. Aquat. Sci.* **70**, 964–972 (2013).

[79] Welch, J. & Jager, H. Thermal Tolerance Metrics for Freshwater Fish, CONUS, Version 1. Oak Ridge National Laboratory (ORNL), Oak Ridge, TN (United States) 10.21951/GMLC_THERMAL_METRIC/1897392 (2022).

[80] Bonacina, L., Fasano, F., Mezzanotte, V. & Fornaroli, R. Effects of water temperature on freshwater macroinvertebrates: a systematic review. *Biol. Rev.* **98**, 191–221 (2023). 36173002 10.1111/brv.12903 PMC10088029

[81] Dahlke, F. T., Wohlrab, S., Butzin, M. & Pörtner, H.-O. Thermal bottlenecks in the life cycle define climate vulnerability of fish. *Science* **369**, 65–70 (2020). 32631888 10.1126/science.aaz3658

[82] Pottier, P. *et al*. Developmental plasticity in thermal tolerance: Ontogenetic variation, persistence, and future directions. *Ecol. Lett.* **25**, 2245–2268 (2022). 36006770 10.1111/ele.14083 PMC9804923

[83] Sasaki, M. *et al*. Greater evolutionary divergence of thermal limits within marine than terrestrial species. *Nat. Clim. Change* **12**, 1175–1180 (2022).

[84] Comte, L. & Olden, J. D. Climatic vulnerability of the world’s freshwater and marine fishes. *Nat. Clim. Change* **7**, 718–722 (2017).

[85] Gunderson, A. R. & Stillman, J. H. Plasticity in thermal tolerance has limited potential to buffer ectotherms from global warming. *Proc. R. Soc. B Biol. Sci.* **282**, 20150401 (2015). 10.1098/rspb.2015.0401 PMC4455808 25994676

[86] Pinsky, M. L., Eikeset, A. M., McCauley, D. J., Payne, J. L. & Sunday, J. M. Greater vulnerability to warming of marine versus terrestrial ectotherms. *Nature* **569**, 108–111 (2019). 31019302 10.1038/s41586-019-1132-4

[87] Barbarossa, V. *et al*. Threats of global warming to the world’s freshwater fishes. *Nat. Commun.* **12**, 1701 (2021). 33723261 10.1038/s41467-021-21655-w PMC7960982

[88] Rohr, J. R. *et al*. The complex drivers of thermal acclimation and breadth in ectotherms. *Ecol. Lett.* **21**, 1425–1439 (2018). 30009486 10.1111/ele.13107

[89] Sasaki, M. & Dam, H. G. Global patterns in copepod thermal tolerance. *J Plankton Res* **43**, 598–609 (2021).

[90] Bates, A. E. *et al*. Geographical range, heat tolerance and invasion success in aquatic species. *Proc. R. Soc. B Biol. Sci.* **280**, 20131958 (2013). 10.1098/rspb.2013.1958 PMC3813329 24266040

[91] Chown, S. L., Duffy, G. A. & Sørensen, J. G. Upper thermal tolerance in aquatic insects. *Curr. Opin. Insect Sci.* **11**, 78–83 (2015). 28285762 10.1016/j.cois.2015.09.012

[92] Rohatgi: Webplotdigitizer: Version 4.5 - Google Scholar. https://apps.automeris.io/wpd4/.

[93] Grosberg, R. K., Vermeij, G. J. & Wainwright, P. C. Biodiversity in water and on land. *Curr. Biol.* **22**, R900–R903 (2012). 23137680 10.1016/j.cub.2012.09.050

[94] Mora, C., Tittensor, D. P., Adl, S., Simpson, A. G. B. & Worm, B. How Many Species Are There on Earth and in the Ocean? *PLOS Biol.* **9**, e1001127 (2011). 21886479 10.1371/journal.pbio.1001127 PMC3160336

[95] Balian, E. V., Segers, H., Lévèque, C. & Martens, K. The Freshwater Animal Diversity Assessment: an overview of the results. *Hydrobiologia* **595**, 627–637 (2008).

[96] Gillmann, S. M., Hering, D. & Lorenz, A. W. Habitat development and species arrival drive succession of the benthic invertebrate community in restored urban streams. *Environ. Sci. Eur.* **35**, 49 (2023).

[97] Nguyen, H. H. *et al*. Stream macroinvertebrate communities in restored and impacted catchments respond differently to climate, land-use, and runoff over a decade. *Sci. Total Environ.* **929**, 172659 (2024). 38657809 10.1016/j.scitotenv.2024.172659

[98] Kunz, S. *et al*. Similarity of stream insect trait profiles across biogeographic regions. *Divers. Distrib.* **30**, e13812 (2024).

[99] Thorne, R. & Williams, P. The response of benthic macroinvertebrates to pollution in developing countries: a multimetric system of bioassessment. *Freshw. Biol.* **37**, 671–686 (1997).

[100] Harzing, A. W. Publish or Perish (2007).

[101] Beck, H. E. *et al*. High-resolution (1 km) Köppen-Geiger maps for 1901–2099 based on constrained CMIP6 projections. *Sci. Data* **10**, 724 (2023). 37872197 10.1038/s41597-023-02549-6 PMC10593765

[102] OpenTreeofLife *et al*. Open Tree of Life Taxonomy (2019).

[103] R Core Team. R: A language and environment for statistical computing. R Foundation for Statistical Computing (2022).

[104] Westgate, M. J. revtools: An R package to support article screening for evidence synthesis. *Res. Synth. Methods* **10**, 606–614 (2019). 31355546 10.1002/jrsm.1374

[105] Wickham, H. *et al*. Welcome to the Tidyverse. *J. Open Source Softw.* **4**, 1686 (2019).

[106] Barrett, T. *et al*. data.table: Extension of ‘data.frame’ (2024).

[107] Michonneau, F., Brown, J. W. & Winter, D. J. rotl: an R package to interact with the Open Tree of Life data. *Methods Ecol. Evol.* **7**, 1476–1481 (2016).

[108] Chamberlain, S. *et al*. taxize: Taxonomic Information from Around the Web (2022).

[109] Pebesma, E. *et al*. sf: Simple Features for R (2024).

[110] Hollister, J. *et al*. elevatr: Access Elevation Data from Various APIs (2023).

[111] Hijmans, R. J., Barbosa, M., Ghosh, A. & Mandel, A. geodata: Download Geographic Data (2024).

[112] Hijmans, R. J., Bivand, R., Dyba, K., Pebesma, E. & Sumner, M. D. terra: Spatial Data Analysis (2024).

[113] Hester, J. *et al*. fs: Cross-Platform File System Operations Based on ‘libuv’ (2024).

[114] Massicotte, P., South, A. & Hufkens, K. rnaturalearth: World Map Data from Natural Earth (2023).

[115] Garnier, S. *et al*. viridis: Colorblind-Friendly Color Maps for R (2024).

[116] Pedersen, T. L. patchwork: The Composer of Plots (2024).

[117] Wilke, C. O. ggridges: Ridgeline Plots in ‘ggplot2’ (2024).

[118] Bayat, H. S. hsbayat/Freshwater_thermtol_db: Freshwater thermal tolerance. *Zenodo*10.5281/zenodo.14056760 (2024).

[119] Verberk, W. C. E. P., Hoefnagel, K. N., Peralta-Maraver, I., Floury, M. & Rezende, E. L. Long-term forecast of thermal mortality with climate warming in riverine amphipods. *Glob. Change Biol.* **29**, 5033–5043 (2023). 10.1111/gcb.16834 37401451

[120] Coutant, C. C. & Pfuderer, H. A. Thermal Effects. *J. Water Pollut. Control Fed.* **45**, 1331–1369 (1973). 4605444

[121] Bornmann, L., Haunschild, R. & Mutz, R. Growth rates of modern science: a latent piecewise growth curve approach to model publication numbers from established and new literature databases. *Humanit. Soc. Sci. Commun.* **8**, 1–15 (2021).38617731

[122] Keck, F., Broadbent, H. & Altermatt, F. Extracting massive ecological data on state and interactions of species using large language models. 2025.01.24.634685 Preprint at 10.1101/2025.01.24.634685 (2025).

[123] Chen, X. & Zhang, X. Large language models streamline automated systematic review: A preliminary study. Preprint at 10.48550/arXiv.2502.15702 (2025).

[124] Tóth, B., Berek, L., Gulácsi, L., Péntek, M. & Zrubka, Z. Automation of systematic reviews of biomedical literature: a scoping review of studies indexed in. *PubMed. Syst. Rev.* **13**, 174 (2024). 38978132 10.1186/s13643-024-02592-3 PMC11229257

[125] Cao, C. *et al*. Automation of Systematic Reviews with Large Language Models. 2025.06.13.25329541 Preprint at 10.1101/2025.06.13.25329541 (2025).

[126] Wu, N. C. *et al*. Reporting guidelines for terrestrial respirometry: Building openness, transparency of metabolic rate and evaporative water loss data. *Comp. Biochem. Physiol. A. Mol. Integr. Physiol.* **296**, 111688 (2024). 38944270 10.1016/j.cbpa.2024.111688

[127] Waldvogel, A.-M., Schreiber, D., Pfenninger, M. & Feldmeyer, B. Climate Change Genomics Calls for Standardized Data Reporting. *Front. Ecol. Evol*. **8** (2020).

[128] Vetter, D., Storch, I. & Bissonette, J. A. Advancing landscape ecology as a science: the need for consistent reporting guidelines. *Landsc. Ecol.* **31**, 469–479 (2016).

